# Clinical Lecture, Chronic Eczema

**Published:** 1907-01

**Authors:** John V. Shoemaker

**Affiliations:** Professor of Materia Medica, Therapeutics, Clinical Medicine and Diseases of the Skin in the Medico-Chirurgical College and Hospital, Philadelphia, Pa.


### TEXAS MEDICAL JOURNAL.

Established July, 1885.

F. E. DANIEL, M. D., - - - - Editor, Publisher and Proprietor.

PUBLISHED MONTHLY.—SUBSCRIPTION $1.00 A YEAR.

VOL. XXII. AUSTIN, JANUARY, 1907. No. 7.

The publisher is not responsible for the views of contributors.

Contributed exclusively to Texas Medical Journal.

### Clinical Lecture, Chronic Eczema.

BY JOHN V. SHOEMAKER, M. D., LL. D.

Professor of Materia Medica, Therapeutics, Clinical Medicine and Diseases of the Skin in the Medico-Chirurgical College and Hospital, Philadelphia, Pa.

Here is a man, age 71, nativity, Ireland, occupation, laborer. About five years ago he had a severe attack of rhus poisoning on his arm and hands. After the poisoning had subsided the skin of his hands remained irritable, itchy and red. A few months later a similar redness and itchy condition of the skin appeared on his legs which soon spread to the thighs and body. The skin of his head and face is also involved as shown by the formation of crusts due to the exudation of serum. He claims that the eruption over his body often itched so severely that his wife had to rub the skin with a coarse towel to give him relief. This severe itching was followed by a weeping condition of the denuded surfaces. The exudate, as he stated, became hard incrustation. No other lesions have been manifested according to his description of the symptoms. His physical condition otherwise is very good, considering his age. His teeth are in a poor condition and evidently he can not properly masticate his food. The tongue is pallid and its papillae are very prominent. The bowels are inclined to be constipated.

A close observation of his skin shows that it is very scaly and infiltrated. It appears infected and feels hot to the touch. There is nothing in his family or personal history that would lead me to suspect contagion.

Before we discuss the disease or give you a diagnosis, we will consider the next patient who is a young woman, age 34, nativity, United States, occupation, dishwasher. Two years ago her hands during the winter months became fissured and bled. She laid the blame to the strong soap she used in washing the dishes and consequently discontinued washing dishes. She sought medical treatment, and received relief, but was not entirely cured. One year ago she was employed again as dishwasher and since then her hands over the entire palm are fissured. The skin is thick and harsh, and looks discolored like the hands of one who immerses them daily in a strong bichloride of mercury solution. Her tongue is also fissured and coated white. She complains of great distress after meals followed occasionally by griping pains and diarrhoea.

The diagnosis of these patients is very clear. The man is suffering from a typical condition known as chronic erythematous eczema. The color of the skin in this patient is subject to variation; at times the color of his face is almost imperceptible to the naked eye, and the subjective symptoms of burning and itching of the skin being the only evidence of the disease; while at other times the color of the skin over his body may vary from a bright red to a violacious hue or even a yellowish complexion, which old erythematous eczema often acquires. Owing to the intense redness, erythematous eczema may be mistaken for erysipelas, but a careful differential diagnosis as outlined in the table on the blackboard will readily assist you in making a correct conclusion:

| *Erythematous Eczema.* | *Erysipelas.* |
|---|---|
| (1) No history of contagion | (1) History of contagion |
| (2) No prodromal symptoms | (2) Prodromal symptoms onset with chill and fever |
| (3) Vesicles occur in patches, if present | (3) Vesicles are discreet |
| (4) Eruption fades imperceptibly into healthy skin | (4) Eruption sharply defined |
| (5) Affected skin is scaly and somewhat infiltrated | (5) Affected skin is glossy and oedematous |
| (6) Color bright or dull red | (6) Color purplish |
| (7) Pain is itching and burning in character | (7) Pain is acute in character |
| (8) Course of disease is chronic | (8) Course of disease is acute |

The diagnosis of the woman's disease is established upon the history and onset. There is no other disease of the skin for which her condition might be mistaken. The characteristic fissures and the infiltrated skin together with the itching and the long standing of the case are amply sufficient to decide at a correct diagnosis.

The pathological condition is followed by cell-proliferation and infiltration. After the eruption has existed for some time, the skin by reason of the interference with the free action of the glands, has a peculiar hard, rough feel, caused by the absence of the normal unctious material and also becomes covered with fine scales. The characteristic redness and sense of warmth of the skin in the acute stage is due to the engorged capillaries. Later there is a rhythmical contraction and dilation of the blood-vessels causing the variation of color so often present in this form of eczema.

It is highly probable that the man's eczema was first brought on by the rhus poisoning and aggravated by his indiscretion of diet and excessive use of alcoholic liquors and tobacco. It is often due to some constitutional disturbances and again it may depend on many local conditions. It is common in diseases of the alimentary system. It is not infrequently a concomitant of disorders of the liver and kidneys. It may also be produced by diseases of the lungs, heart and blood-vessels. It may be excited by gout, rheumatism, diabetes, affections of the nervous system or sexual apparatus, or it may be followed by a fever. Eczema may be awakened by external irritation as in this case by the rhus poison, dyes, atmospheric changes, excessive use of water, strong soaps, acids, alkalies, turpentine, croton oil, tartar emetics, cantharides, and a long list of irritant substances some of which are, and others are not, used in medicine.

In the woman's case the original cause of the fissure was due to the water and strong soap used in washing dishes.

It follows from these facts that the treatment must vary according to the cause of the eczema. Especially is it important to establish the origin in the chronic form. In many cases constitutional treatment is of more value than local applications. Internally we will give the man or first patient a capsule containing creosote (beetchwood], m. ii, and

℞ Strychnine sulphatis ................gr. 1-60

Podophyllotoxin ....................gr. 1-8

Olei menthae piperitae ... .........m 1-20

Salicini ..........................gr. iii

Misce, fiant pilula No. xxx.

Sig. One pill after each meal and one at bed time.

Externally, he needs an emollient to keep the skin soft and piable and one that will also have a stimulating influence upon the affected parts. I know of no better combination that will help this man more or even as much, as an ounce of oil of cade to five ounces of cod liver oil.

In endeavoring to cure the woman's fissured eczema, stimulating ointment will probably do more for her than internal remedies. However, it would be well to give her some remedy to correct her disturbed digestion and correct her torpid liver. Internally the following combination will be of value to her:

℞ Acidi nitrohydrochlorici dilute ..........oz. ss
Glycerite pepsini (nat. for.) qs. ad......oz. vi.

M. Sig.: Two teaspoonfuls in two tablespoonfuls of water after each meal.

Externally, I have found the following ointment to do well in similar cases:

℞ Olei cadini ............................dr. ii
Acidi Salicylatis ..........................dr. i
Unguenti hydraryri nitritis ................ozs. ss
Sulphuris sublamati .....................dr. ii
Unguenti aquae rosae.....................oz. ss

M. Ft. unguentum.

Sig.: Apply locally to the palms of the hand three times daily.

We must also request her and the man to eat simple and well-cooked foods. No pastries or fried foods. Too much of the farinaceous foods is likewise interdicted.

